# Supplementary material for: Animal trait variation at the within-individual level: erythrocyte size variation and malaria infection in a tropical lizard
Source: PeerJ. 2022 Feb 23;10:e12761. doi: 10.7717/peerj.12761 (PMC8881909; doi:10.7717/peerj.12761)
Supplement: Supplemental Information 1 [file peerj-10-12761-s001.zip › code_data_wiv_erysize/wiv_erythrocite_analysis.pdf]

WIV in a Tropical Lizard: Code and Results

Virnaliz Cruz

```r
# Colorblind friendly palette
cbPalette <- c("#56B4E9","#CC79A7", "#009E73",
               "#F0E442", "#0072B2", "#D55E00")
```

Note:Data AND Rmd file should be in same folder

Model1. BCI predicted by infection status, sex and season

```
##
## Call:
## glm(formula = BCI ~ infection.status + Sex + Season, data = matureErythrocyteSeasonalDF)
##
## Deviance Residuals:
##      Min        1Q    Median        3Q       Max
## -0.23124  -0.04011  -0.00155   0.03609   0.32691
##
## Coefficients:
##                   Estimate Std. Error t value Pr(>|t|)
## (Intercept)       -0.02269    0.01225  -1.852   0.0656 .
## infection.status1  0.01491    0.01009   1.478   0.1410
## SexM               0.02990    0.01162   2.574   0.0108 *
## Seasonwinter      -0.01515    0.01012  -1.497   0.1361
## ---
## Signif. codes:  0 '***' 0.001 '**' 0.01 '*' 0.05 '.' 0.1 ' ' 1
##
## (Dispersion parameter for gaussian family taken to be 0.004883579)
##
##     Null deviance: 1.03890  on 201  degrees of freedom
## Residual deviance: 0.96695  on 198  degrees of freedom
## AIC: -495.81
##
## Number of Fisher Scoring iterations: 2
```

Table 1: Predicted body condition index (BCI) values by infection status

infection.status	Sex	Season	Predicted	lower	upper	se.predict
0	F	summer	-0.0226911	-0.0467107	0.0013286	0.0122549
1	F	summer	-0.0077819	-0.0328985	0.0173348	0.0128146
0	M	summer	0.0072132	-0.0106057	0.0250322	0.0090913
1	M	summer	0.0221224	0.0060863	0.0381585	0.0081817

infection.status	Sex	Season	Predicted	lower	upper	se.predict
0	F	winter	-0.0378365	-0.0595682	-0.0161047	0.0110876
1	F	winter	-0.0229273	-0.0484013	0.0025468	0.0129970
0	M	winter	-0.0079322	-0.0260149	0.0101505	0.0092259
1	M	winter	0.0069770	-0.0127557	0.0267098	0.0100677

Figure 1

Predicted partial relationships between infection status and body condition index (BCI) for female and male *A.gundlachi* during the A) 2015 summer and B) 2016 winter season. Grey error bars represent 95 % CI and black error bars represent one standard error.

```
# Saving BCI plot as pdf
pdf('bcipredictedinfection.pdf')
grid.arrange(summerBCIplot2, winterBCIplot2 , ncol = 2)
dev.off()
```

```
## pdf
##   2
```

```
grid.arrange(summerBCIplot2, winterBCIplot2, ncol = 2)
```

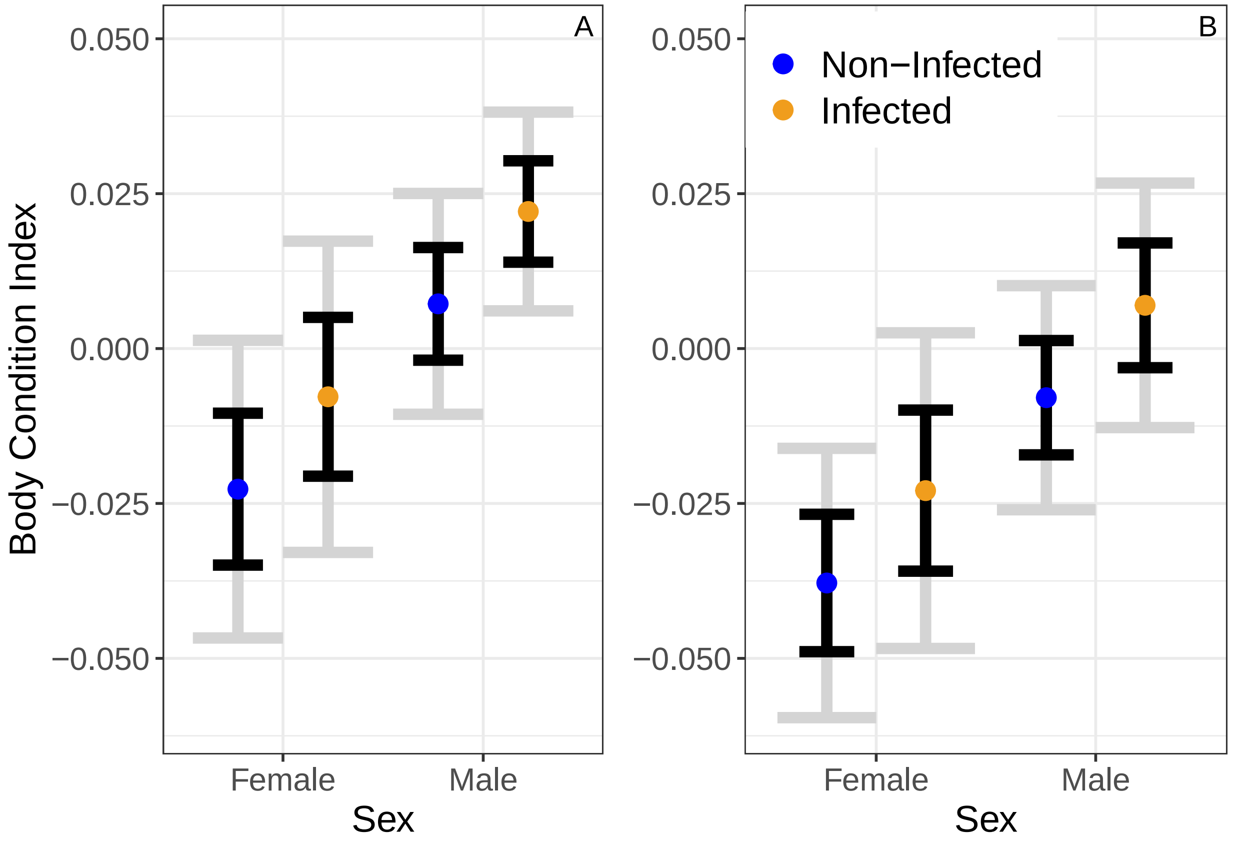

Model2. Probability of infection predicted by CV, season, svl and sex

```
##
## Call:
## glm(formula = infection.status ~ CV + Season + Sex + SVL, family = binomial(logit),
##     data = matureErythrocyteSeasonalDF)
##
## Deviance Residuals:
##     Min       1Q   Median       3Q      Max
## -1.8770  -0.9641  -0.5657   1.0129   1.9958
##
## Coefficients:
##              Estimate Std. Error z value Pr(>|z|)
## (Intercept)  -7.56809    1.68860  -4.482  7.4e-06 ***
## CV            0.35521    0.12154   2.923 0.003472 **
## Seasonwinter -0.46096    0.31255  -1.475 0.140259
## SexM         -0.23213    0.44788  -0.518 0.604269
## SVL           0.07271    0.02203   3.300 0.000967 ***
## ---
## Signif. codes:  0 '***' 0.001 '**' 0.01 '*' 0.05 '.' 0.1 ' ' 1
##
## (Dispersion parameter for binomial family taken to be 1)
##
##     Null deviance: 279.32  on 201  degrees of freedom
## Residual deviance: 246.12  on 197  degrees of freedom
## AIC: 256.12
##
## Number of Fisher Scoring iterations: 4
```

Table 2: Predicted probability of infection by the coefficient of variation (CV)

genCV	Sex	Season	SVL	predPI	lower	upper	se.predict
8.000000	M	winter	56.5	0.2122183	0.0695361	0.3549006	0.0727971
8.098361	M	winter	56.5	0.2181182	0.0757800	0.3604563	0.0726215
8.196721	M	winter	56.5	0.2241354	0.0822305	0.3660402	0.0724004
8.295082	M	winter	56.5	0.2302697	0.0888852	0.3716542	0.0721350
8.393443	M	winter	56.5	0.2365207	0.0957408	0.3773006	0.0718265
8.491803	M	winter	56.5	0.2428879	0.1027933	0.3829824	0.0714768
8.590164	M	winter	56.5	0.2493705	0.1100378	0.3887031	0.0710881
8.688525	M	winter	56.5	0.2559676	0.1174684	0.3944668	0.0706629
8.786885	M	winter	56.5	0.2626781	0.1250781	0.4002782	0.0702041
8.885246	M	winter	56.5	0.2695009	0.1328589	0.4061429	0.0697153
8.983607	M	winter	56.5	0.2764344	0.1408018	0.4120671	0.0692003
9.081967	M	winter	56.5	0.2834771	0.1488967	0.4180576	0.0686635
9.180328	M	winter	56.5	0.2906272	0.1571322	0.4241222	0.0681097
9.278688	M	winter	56.5	0.2978826	0.1654958	0.4302695	0.0675443
9.377049	M	winter	56.5	0.3052412	0.1739740	0.4365085	0.0669731
9.475410	M	winter	56.5	0.3127007	0.1825520	0.4428494	0.0664024
9.573771	M	winter	56.5	0.3202584	0.1912141	0.4493027	0.0658389
9.672131	M	winter	56.5	0.3279116	0.1999434	0.4558798	0.0652899
9.770492	M	winter	56.5	0.3356574	0.2087223	0.4625924	0.0647628

genCV	Sex	Season	SVL	predPI	lower	upper	se.predict
9.868852	M	winter	56.5	0.3434926	0.2175324	0.4694529	0.0642654

Figure 2

Predicted relationship between the probability of infection and coefficient of variation (CV) and for female and male *A.gundlachi* during the A) 2015 summer season and B) 2016 winter season. Points represent *A.gundlachi* individual data relating infection status to the coefficient of variation (CV) by snout to vent length (SVL).

```
## Warning: It is deprecated to specify ‘guide = FALSE‘ to remove a guide. Please
## use ‘guide = "none"‘ instead.
```

```
## pdf
##   2
```

```
## Warning: It is deprecated to specify ‘guide = FALSE‘ to remove a guide. Please
## use ‘guide = "none"‘ instead.
```

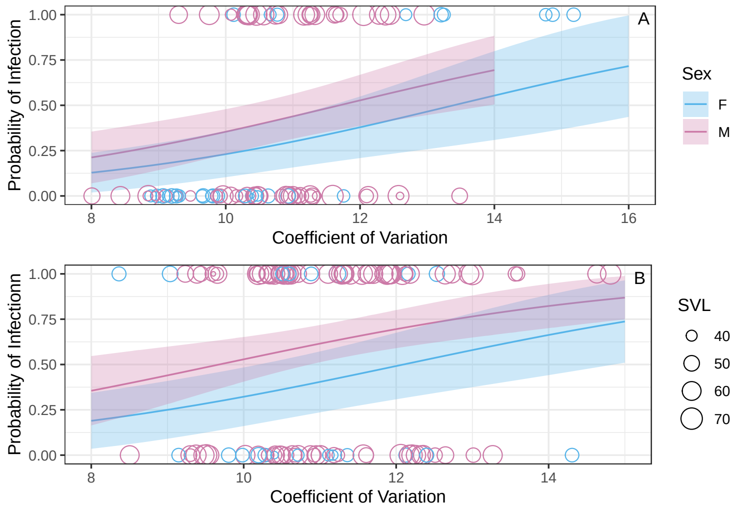

Females and males of both summer and winter season displayed an increasing relationship between the probability of infection and the coefficient of variation of erythrocytes. SVL had an estimate of 0.072 $\pm$0.02, while CV has an estimate of 0.36 $\pm$0.12.

Model3. Body condition index (BCI) predicted by CV, season, and sex

```
##
## Call:
## glm(formula = BCI ~ CV + Season + Sex, data = matureErythrocyteSeasonalDF)
##
## Deviance Residuals:
##      Min        1Q    Median        3Q       Max
## -0.23738  -0.03715  -0.00241   0.03429   0.33211
##
```

```
## Coefficients:
##                Estimate Std. Error t value Pr(>|t|)
## (Intercept)  -0.0081175  0.0430876  -0.188  0.85076
## CV           -0.0007423  0.0037830  -0.196  0.84464
## Seasonwinter -0.0176958  0.0101050  -1.751  0.08146 .
## SexM          0.0321625  0.0115914   2.775  0.00605 **
## ---
## Signif. codes:  0 '***' 0.001 '**' 0.01 '*' 0.05 '.' 0.1 ' ' 1
##
## (Dispersion parameter for gaussian family taken to be 0.004936494)
##
##     Null deviance: 1.03890  on 201  degrees of freedom
## Residual deviance: 0.97743  on 198  degrees of freedom
## AIC: -493.63
##
## Number of Fisher Scoring iterations: 2
```

Figure 3

Predicted relationship between the coefficient of variation (CV) and body condition index (BCI) for female and male *A.gundlachi* during the A) 2015 summer season and B) 2016 winter season. Points represent *A.gundlachi* individual data relating the coefficient of variation (CV) to body condition index (BCI).

```
## Scale for 'fill' is already present. Adding another scale for 'fill', which
## will replace the existing scale.
## Scale for 'fill' is already present. Adding another scale for 'fill', which
## will replace the existing scale.
```

```
## Scale for 'colour' is already present. Adding another scale for 'colour',
## which will replace the existing scale.
```

```
## pdf
##   2
```

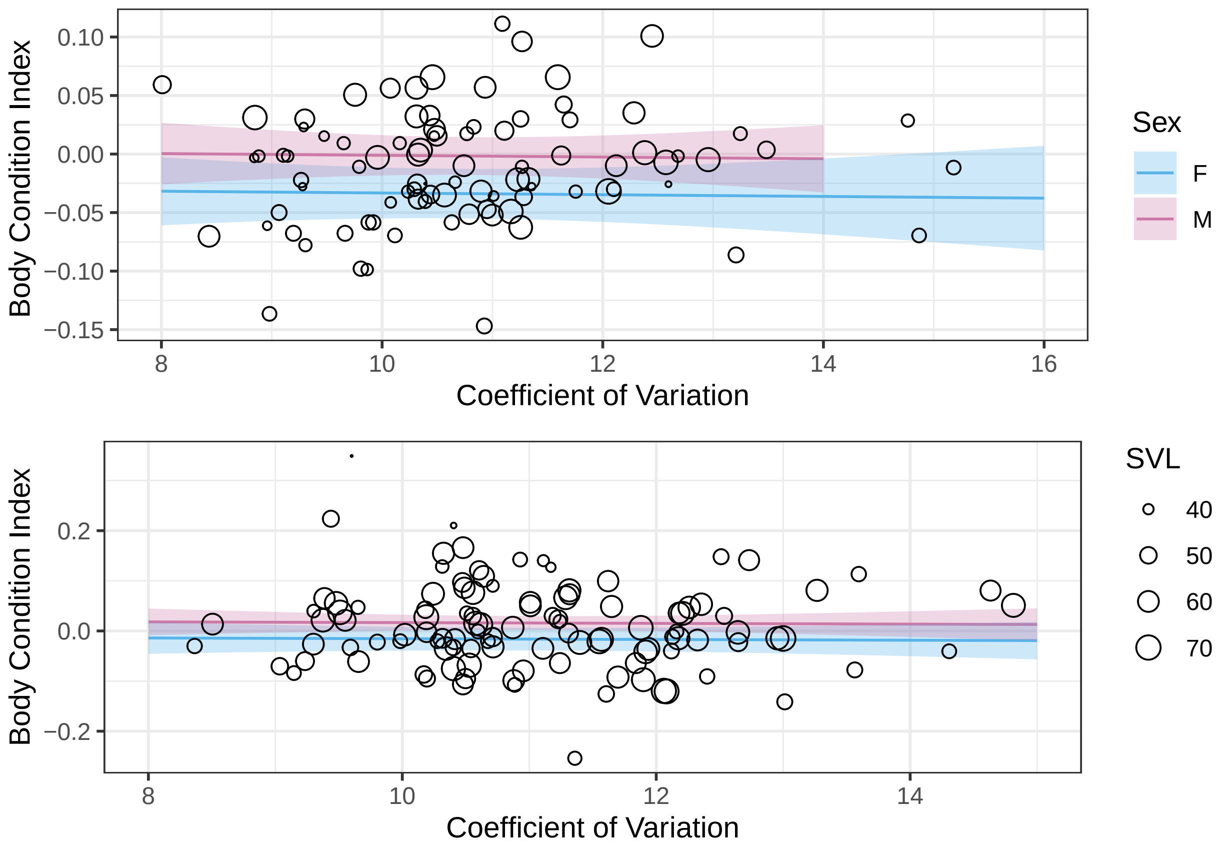
Body Condition Index
Coefficient of Variation
Sex
F
M
Body Condition Index
Coefficient of Variation
SVL
40
50
60
70
